# A Randomized Controlled Trial Evaluating the FIT Game’s Efficacy in Increasing Fruit and Vegetable Consumption

**DOI:** 10.3390/nu13082646

**Published:** 2021-07-30

**Authors:** Heidi J. Wengreen, Damon Joyner, Sheryl S. Kimball, Sarah Schwartz, Gregory J. Madden

**Affiliations:** 1 Department of Nutrition Dietetics and Food Sciences, Utah State University, Logan, UT 84322, USA; rd4you2@gmail.com; 2 Department of Exercise and Nutrition Sciences, Weber State University, Ogden, UT 84408, USA; damonjoyner@weber.edu; 3 Department of Psychology, Utah State University, Logan, UT 84322, USA; sarah.schwartz@usu.edu (S.S.); greg.madden@usu.edu (G.J.M.)

**Keywords:** consumption, incentive, gamification, fruit, vegetable, healthy eating

## Abstract

Few children eat the recommended amounts of fruits and vegetables (FV). Although incentive-based interventions can increase FV consumption, this approach is costly and may be viewed as controversial due to the possible negative effects on intrinsic motivation. The FIT Game was designed to address these challenges. Four elementary schools were randomly assigned to either cooperatively play the FIT Game (*n* = 881) for ~8 weeks or to a no-game Control condition (*n* = 978). The FIT Game was presented daily as comic-book formatted episodes projected onto a large screen in the school cafeteria throughout lunchtime. All children could see the episodes which communicated daily whole-school vegetable-eating goals and illustrated the progress of the game’s heroes when these goals were collectively met. Photo estimates of FV consumption and skin carotenoid concentrations (biomarker of carotenoid consumption) were collected at baseline, during the last 5 days of the FIT Game, and 3 months after the intervention concluded. Control schools followed the same FV consumption-monitoring procedures for the same duration. At the conclusion of the intervention phase, children attending the FIT Game schools consumed more vegetables (*d* = 0.41), more fruit (*d* = 0.39), and had higher skin carotenoids (*d* = 0.66) than at baseline. These statistically significant increases were maintained at a 3-month follow-up for vegetables (*d* = 0.21, the food targeted for change) and carotenoids (*d* = 0.53). Thus, the no-cost virtual incentives of the FIT Game increased FV consumption in the short- and long-run, without negatively impacting intrinsic motivation.

## 1. Introduction

Adherence to a diet rich in fruit and vegetables (FV) can decrease the risk of hypertension, coronary heart disease, stroke, some types of cancer, premature mortality, and may prevent weight gain [1,2,3,4]. Despite these benefits, most US children do not consume the recommended daily amounts of these foods. This behavior is still less prevalent among children living in poverty [5,6,7]. This portends poorly for later health, particularly in light of decreasing FV consumption as children age into adolescence and beyond [8,9].

US public schools are an ideal location for impacting healthy eating. Here, nearly 30 million children are served lunches that can include FV, if they choose them [10]. However, systematic reviews and meta-analyses of school-based studies that objectively measure FV consumption (e.g., plate waste measures) reveal mixed outcomes. Multi-component interventions can significantly increase fruit consumption, but vegetable consumption has been more difficult to impact [11]. 

The present research took an instrumental-learning habit-formation approach to increasing adaptive behavior [12,13,14]. That is, desired behaviors are increased by awarding contingent incentives, and by repeating this response-reinforcer sequence in the same context (e.g., lunchtime in the school cafeteria) the desired response can transition from incentive-directed behavior to a more automatic, context-evoked behavior [15,16]. This incentive-based habit-formation approach has improved health-impacting behaviors in adults [14]. In children, studies arranging tangible incentives (e.g., toys, tokens or money) for consuming more FV have produced positive short-term effects [17] and, when an extended-duration intervention is implemented in the same context (e.g., the school cafeteria), beneficial long-term effects, even after the incentives are withdrawn [18,19,20,21,22]. 

Incentive-based approaches are not without shortcomings; three are noted here. First, incentive costs may be prohibitive for public schools, particularly those in lower-income regions. Second, incentives may induce children to cheat [17]. Third, incentivizing a self-interested behavior (healthy eating) runs counter to psychological research demonstrating that, under some circumstances, incentives can displace intrinsic motivation to engage in the desired behavior [23,24,25,26]. Applied to school-based healthy eating interventions, if incentives at school decrease intrinsic motivation, then children should consume fewer FV at home, where incentives are unavailable. In addition, when incentives are withdrawn, FV consumption should decrease *below* pre-intervention levels. 

The FIT Game was developed to address two of these shortcomings [27]. First, no-cost virtual incentives (existing only within a science-fiction game) replace tangible incentives, thereby reducing operating costs. Second, to discourage cheating, the virtual incentives are awarded to characters in the game narrative, not to individuals at school. Reducing individual benefit while leaving the risk of getting caught unchanged is known to reduce cheating [28]. 

The third shortcoming—concerns about intrinsic motivation—is not explicitly addressed by the FIT Game, nor has it been evaluated in prior studies [27,29,30,31]. The present study fills this gap in the literature. First, FV consumption was reassessed 3 months after the FIT Game’s virtual incentives were removed to see if they reduce intrinsic motivation to eat these foods. Second, skin carotenoids (a validated biomarker of FV consumption in children [32]) were monitored throughout the study to see if incentives decreased intrinsic motivation to eat FV at home. If the latter occurred, it would be revealed by a pattern of increased FV consumption in at-school measures but unchanged carotenoid levels. 

Because habit formation occurs when instrumental behavior is reinforced repeatedly in the same context [14,33], the FIT Game was played for an extended duration, without interruption. This necessitated a different experimental design than the within-schools reversal-design used in prior evaluations of the FIT Game [27,29,30,31]. Thus, the present experiment is the first to assess the efficacy of the FIT Game using a randomized controlled trial design, which allowed children to uninterruptedly play the game for ~8 weeks. We hypothesized that (1) vegetable consumption and skin-carotenoid levels would be significantly higher among children attending the FIT Game schools and (2) these increases would be maintained at the 3-month follow-up. 

## 2. Methods

Participants were school children in kindergarten through fifth-grade (ages 5–11) attending one of four public elementary schools in Logan, UT, USA. A power analysis (based on the estimated effect size and variance observed in previous FIT Game studies [27,29,30]) indicated that recruiting four schools with at least 400 children per school would provide >90% power to detect a 30% within-subjects increase in vegetable consumption. Four of the city’s six elementary schools were selected due to the comparable percentages of children that qualified for free or reduced-price lunch (a measure of socioeconomic status; see Table 1). In recruitment meetings, the study personnel described the goals and planned activities to the principal, who consulted with teachers and parent-teacher organizations. All four of the schools recruited volunteered to participate. 

The experiment was conducted during the 2016/2017 and 2017/2018 academic years; the intervention occurred in October–December, and the follow-up occurred in March. In each year, two schools were randomly assigned to the FIT Game or Control group using the flip of a two-sided coin.

A passive, opt-out consent was provided to all students’ parent/guardians with a description of the FIT Game and the ways in which FV consumption would be measured in the school cafeteria (food waste and tray photos). In the FIT Game schools, 39 of 920 children (4%) opted out of participation; 32 of 1010 children (3%) opted out of the Control procedures. A separate, active-consent form was provided to parents/guardians inviting their children to complete a demographic survey and to have their height, weight, and skin-carotenoid concentration assessed at school. For the latter measures, fewer parents provided active consent: 343 (39%) and 395 (40%) children in the FIT Game and Control schools, respectively. The research protocol, including the passive and active consent procedures, was reviewed and approved by the Institutional Review Board at Utah State University (protocol #10287). Throughout the study, the cafeterias adhered to their pre-planned menus, following the US National School Lunch Program guidelines (at least one fruit and one vegetable option offered each day; children are not required to take either). The schools served predominantly pre-packaged, canned or frozen FV; they did not change offerings based on seasonal availability. 

### 2.1. Baseline Phase

In all four schools, daily FV consumption of all the children eating school-prepared lunch was estimated using a waste-based measure. Qualifying daily FV prepared was weighed by the cafeteria staff before lunch; nonqualifying FV (potatoes, vegetables in soups, ketchup, and fruit juice) were not weighed. From this value, the weights of FV not served plus the amount collected in separate fruit and vegetable waste bins was subtracted (supervised by the study personnel). The difference provided a daily estimate of FV consumption which was used to evaluate the stability of consumption over time (baseline phase) and to determine if daily vegetable-consumption goals were met in the intervention phase (see below). Periodic errors in weighing FV prepared and not served rendered these estimates insufficiently accurate to serve as a dependent measure. 

When consumption stabilized over time in the baseline phase (no monotonic upward or downward trend over 3 days), the study personnel took before/after digital photos of each child’s lunch tray for the next 5 days. Photos were not taken of lunches brought from home. “Before” photos were taken as the child exited the cafeteria line with a full plate of food, and “after” photos were taken after they had eaten lunch and approached the waste bins. Student-identification (ID) numbers were recorded with the photo, and were used to pair the before and after photos. When children occasionally asked why photos were being taken, the study personnel indicated that they were interested in learning what foods children like to eat. To ensure that these procedures did not influence the study outcomes, the photo procedures were implemented identically in all schools. Photos were later coded and used as the dependent measure of FV consumption at school; see Data Preparation section below. 

For children whose parent/guardian signed the opt-in consent form, baseline measures of height (shoes off), weight (clothes on, shoes and coats off), and skin-carotenoid concentrations were collected by the trained study personnel. Two Pharmanex BioPhotonic Scanners (Nu Skin) were used to assess skin carotenoid concentrations. The scanners use resonance Raman spectroscopy applied to the palm of the hand for ~45 s. Raman counts can range from 0 to 70,000. Each child was scanned twice and, if the scores differed by >2000, a third scan was taken. The two scores that were within 2000 were averaged and recorded. Scanners were calibrated daily according to the manufacturer’s specifications. Reassessments conducted later in the study were done using the same scanner as was used for that child during the baseline phase.

### 2.2. Intervention Phase

The sections that follow outline group-specific procedures used during the intervention phase. In the final 5 days of this phase, before/after lunch-tray photos were taken and height, weight, and skin carotenoid concentrations were assessed in all the schools, just as during the baseline phase. 

#### 2.2.1. FIT Game Schools

**Figure 1:**
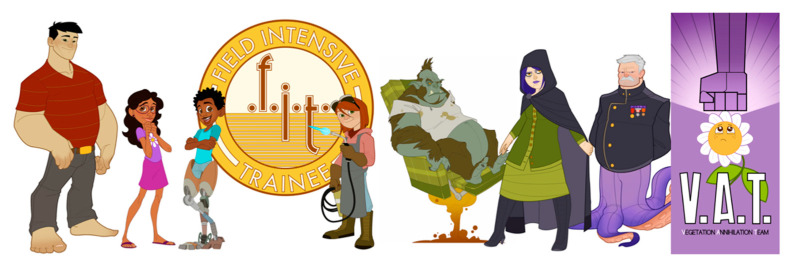
The four heroic members of the field intensive trainees (FITs) and the three villainous members of the vegetation annihilation team (VAT). Reprinted with permission from Utah State University. Copyright 2017 and 2021, Utah State University.

At the beginning of Phase 2, a school assembly was held in the FIT Game schools. At the assembly, children were introduced to the heroes of the FIT Game—the Field Intensive Trainees (FITs)—and the villains of the game—the Vegetation Annihilation Team (VAT; see Figure 1). The game narrative, developed in collaboration with Schell Games (Pittsburgh, PA, USA), was then introduced—to stop the VAT from doing evil, the FITs must find and capture the three VAT leaders. Children at the assembly were informed that since the FITs were trainees, they would need a school to help them do this. The way to help was to eat more vegetables in the school cafeteria. Vegetable consumption was targeted for improvement since it is more difficult to change than fruit consumption [11,34,35,36], thereby providing a rigorous test of the efficacy of the FIT Game.

**Figure 2:**
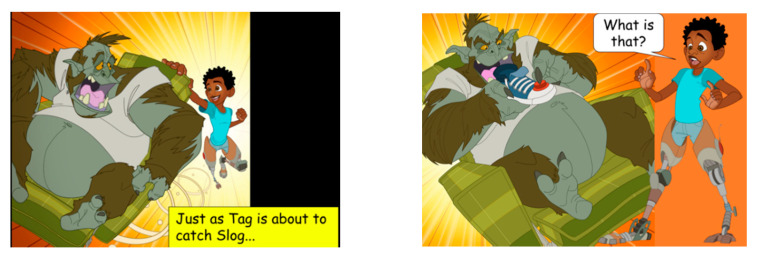
Two sequential slides of a FIT Game episode. The FIT hero, Tag, is about to capture a villain, Slog, in the first slide, but in the second, Slog uses a sneaker-shaped transporter device. Reprinted with permission from Utah State University. Copyright 2017 Utah State University.

The next day, and throughout the remainder of the intervention phase, a FIT Game episode was displayed on a large screen in the cafeteria. Each comic-book formatted episode (~3 min) was composed of a series of self-advancing slides. In each slide, a still picture of the characters, speech bubbles, setting, action, etc. was displayed (see Figure 2). Episodes looped continuously throughout the lunch period. Daily episodes typically ended with a cliff-hanger ending and a request that the children eat a little more vegetables than normal, even if that is just one bite. 

New episodes were presented in the cafeteria on day X contingent upon waste-estimated vegetable consumption on day X−1. The daily vegetable-consumption goal was always to consume at or above the 55th percentile of the school’s own lunchtime vegetable consumption over the previous 10 days. This goal-setting algorithm was used daily to update the goal [37]. In this way, the goal was gradually increased when the goal was consistently met, and gradually decreased if the school consistently failed to meet the goal. Children were not informed how vegetable-consumption goals were calculated; the characters in the episodes simply encouraged them to eat a few more bites than normal. When goals were not met, the new “episode” showed one FIT character whose speech bubbles reminded them of events from the prior episode and encouraged the children to eat more vegetables. 

When vegetable consumption exceeded the daily goal, the school was awarded an amount of in-game currency (FIT points) proportional to the amount in which the goal was exceeded. FIT points were periodically needed within the episodes to purchase items (e.g., a gift for a giant worm guarding the entrance to a wormhole). When these items were needed, children voted in the cafeteria on what item should be purchased. 

Each new episode continued the good vs. evil narrative, culminating in a “boss battle” between the FITs and the leader of the VAT. A total of 32 episodes were presented (available upon request from the corresponding author). In total, the FIT Game schools played the game for 44 days in year 1 (12 days in which the vegetable-eating goal was not achieved) and 39 days in year 2 (7 days without meeting the goal). 

#### 2.2.2. Control Schools

No intervention was provided during this phase in the Control schools, but FV waste was collected daily, exactly as in the FIT Game schools. 

### 2.3. Three-Month Follow-Up

Three months after the FIT Game ended, the study personnel returned to all four schools and collected FV consumption data for 5 consecutive days using the food-waste and before/after tray photo methods as outlined above. In addition, follow-up assessments of height, weight, and skin carotenoid concentrations were recorded. A 3-month interval was selected since it provided sufficient time for skin carotenoid levels to return to baseline levels if FV consumption did the same [38].

### 2.4. Data Preparation

The coding of lunch tray photos followed our previously published methods [19]. Briefly, the weight of a cup of each vegetable and each fruit offered in the cafeteria was recorded on days when tray photos were collected. At the conclusion of the study, two trained observers (blinded to the group assignment and phase) independently coded each pre- and post-lunch tray photo, recording the amount of each FV consumed. Mixed FV items (e.g., vegetable soup) were not included in the analysis. The scale used ranged from 0 to 1 cups in 0.123-cup (1/8th-cup) increments. The mean of the two estimations was taken as the final estimate unless the observers disagreed by >0.125 cup. When this happened, a third observer (blinded as above) independently coded the photos. If the third observer’s estimate did not match either of the other two, a registered dietitian was to code the photo pair to make the final estimation (this step was never necessary). 

### 2.5. Statistical Analyses

All the analysis were conducted in R 4.1.0 [39] and a significance level of 0.05 was used. Full code, data, and output are available on Open Science Framework at doi: 10.17605/OSF.IO/GVPQS. The distribution of children’s vegetable and fruit consumption and skin carotenoid scores were examined for normality. The fruit and vegetable intake distributions showed significant skewness and kurtosis consistent with zero-inflated distributions (43% and 19% of the children consumed no vegetables and no fruit, respectively, during the baseline phase). However, the large sample sizes provide some protection against this violation of the model assumption. 

To account for the non-independence of repeated observations (micro-units of level 1) nested within the child (meso-units of level 2) and further nested within school (macro-units of level 3), multilevel models (MLM) were fit separately for each of the dependent variables (DV): Vegetable consumption (Model 1), fruit consumption (Model 2), combined FV consumption (Model 3), and skin carotenoid concentration (Model 4). MLM, also referred to as mixed effects regression, appropriately model independent variables (IV) or predictors at any/all of the levels via fixed effects and partition variance in the DV between the nesting levels via random effects [40]. Benefits of MLM over similar analysis of variance (ANOVA) based models include: Ability to model more than two levels, no reliance on the assumptions of homogeneity of variance (HOV) and sphericity, and the ability to include students with partial data (only one or two time points) [41]. 

All MLMs were optimized with restricted maximum likelihood, random intercepts, and fit via the ‘lme4’ package [42]. Each MLM assessed if the intervention moderated change in the corresponding DV over the three phases of the intervention, while controlling for grade and the hierarchical data structure. Model 4 was additionally able to control for grade, sex, race/ethnic group, baseline body mass index, and child’s exposure to second hand smoke in their home. 

The overall quality of model fit was assessed through various *R*^2^ measures available in the ‘performance’ package [43], Nakagawa’s [44] method for marginal (fixed effects only) and conditional (fixed and random effects) variance explained, and the level-specific variance reduction as detailed by Hox [41]. Significance of fixed model parameter estimates was measured with Wald t-tests utilizing Satterthwaite’s method for degrees of freedom, whereas Likelihood Ratio Tests for single term deletion were used for the evaluation of significance of estimated variance components (i.e., random effects). 

Significant interactions between intervention and phase were probed with follow-up pairwise comparison of the estimated marginal means, both within (Tukey’s HSD adjustment) and between groups (no adjustment), including calculating Cohen’s d-like standardized mean difference (SMD) effect sizes. These effect sizes were standardized with the standard deviation of all baseline measures for all the students from all four schools.

## 3. Results

No formal measures of student engagement with the FIT Game episodes were collected. However, children were frequently observed by the study personnel to point at the screen, react to events happening within the episodes, and were overheard talking about these events while at the waste station. During the baseline phase, the two groups consumed comparable amounts of vegetables (*p* = 0.433) and had very similar skin carotenoid concentrations (*p* = 0.823). However, baseline fruit consumption was significantly higher in the control schools; a difference of 4.97 g (*F*_(1,1450)_ = 5.92, *p* = 0.015).

All four MLM models converged under the default optimizer with intended fixed and random effects. Table 2 specifies all parameter estimates, measures of fit, and corresponding sample sizes. Of note, the incorporation of the nesting structure (random effects) accounted for a large proportion of the variance in each DV (compare marginal and conditional *R*^2^ values) and all variance components were significant. For all DVs there was a significant interaction between the intervention and phase, despite the main effect for the FIT Game intervention being non-significant. 

**Figure 3:**
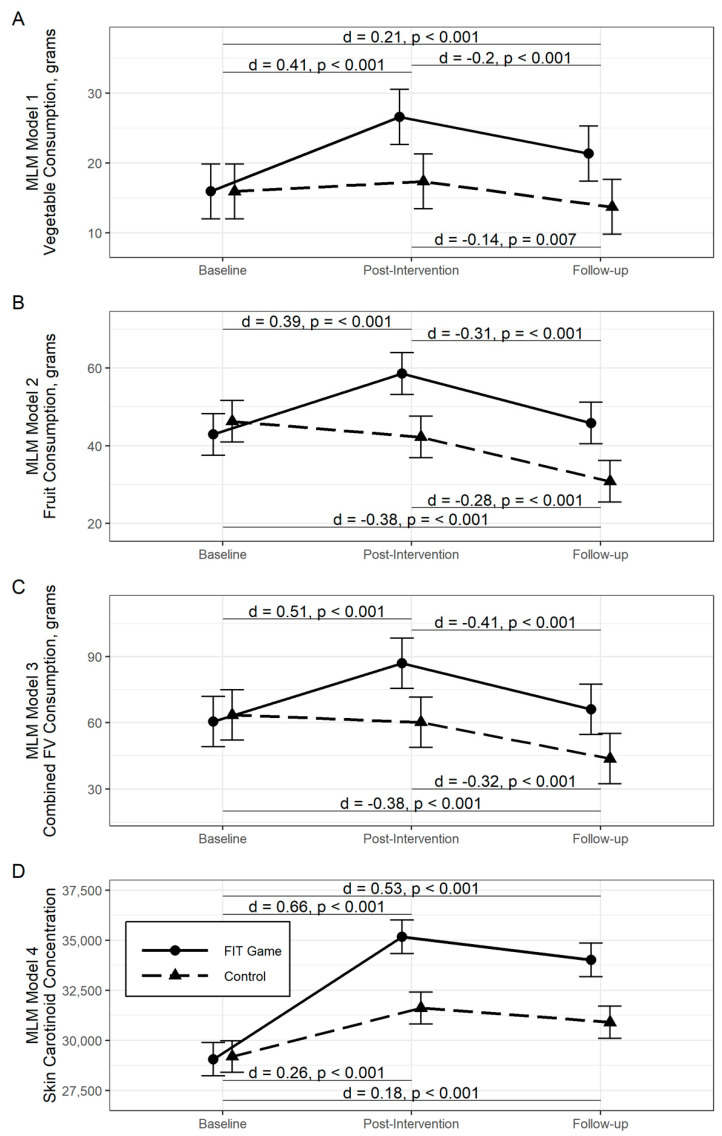
Estimated marginal mean (±SEM) for separate multilevel models (MLM) for consumption (in grams) of vegetables, fruit, and combined fruit and vegetable (FV), as well as skin carotenoid levels in FIT Game and Control schools across the three phases of the experiment. Significant (α = 0.05, Tukey’s HSD) within-group differences are indicated with Cohen’s d-like standardized mean difference (SMD) effect sizes.

Panels A–D of Figure 3 show the marginal mean grams (given in Table 3) of vegetables, fruits, and combined FV consumed, and skin carotenoid concentrations, respectively for each group across the three phases of this study, as estimated by the four separate MLM. Significant within-group comparisons are noted and effect sizes given where applicable. Estimated marginal means and SEM values are provided in Table 3 along with between-group comparisons. 

At the end of the intervention phase, children attending the FIT Game schools were consuming significantly more vegetables than during baseline (∆*M_BL-PI_* = 10.66 g, *d* = 0.41, *p* < 0.001). Although children attending the Control schools started at a similar consumption level, no such within-group gain was observed (∆*M_BL-PI_* = 1.43 g, *d* = 0.06, *p* = 0.458). At the follow-up assessment, nearly half of the gains measured for vegetable consumption among the children attending the FIT Game schools was lost (∆*M_PI-FU_* = −5.25 g, *d* = −0.20, *p* < 0.001), however a moderate long-term increase above the baseline was established (∆*M_BL-FU_* = 5.41 g, *d* = 0.21, *p* < 0.001). Conversely, a moderate long-term reduction in vegetable consumption was observed for children attending the Control schools (∆*M_PI-FU_* = −3.65 g, *d* = −0.14, *p* = 0.007).

Although not targeted for change by the intervention, at the end of the intervention phase children attending the FIT Game schools also consumed significantly more fruit than during the baseline (∆*M_BL-PI_* = 15.66 g, *d* = 0.39, *p* < 0.001). However, this relatively large short-term gain washed out by the end of the follow-up period (∆*M_PI-FU_* = −12.72 g, *d* = −0.31, *p* < 0.001), so fruit consumption returned to the pre-intervention level (∆*M_BL-FU_* = 2.95 g, *d* = 0.07, *p* = 0.332). Unexpectedly, a small but steady long-term decline in fruit consumption was observed among the students attending the Control schools (∆*M_BL-FU_* = −15.47 g, *d* = −0.38, *p* < 0.001).

When combined, the amount of FV consumed by children attending the FIT Game schools similarly exhibited a short-term increase (∆*M_BL-PI_* = 26.45 g, *d* = 0.51, *p* < 0.001) that was all but lost at follow-up (∆*M_BL-FU_* = 5.53 g, *d* = 0.11, *p* = 0.075). Echoing the fruit only consumption, the students attending the Control schools showed a small but steady decline in total FV consumed (∆*M_BL-FU_* = −10.70 g, *d* = 0.38, *p* < 0.001).

The largest and longest lasting gains were measured in skin carotenoid concentrations. Not only did children attending the FIT Game schools have much higher levels at the end of the intervention (∆*M_BL-PI_* = 6101, *d* = 0.66, *p* < 0.001), but this increase was maintained at the 3-month follow-up (∆*M_BL-FU_* =4951, *d* = 0.53, *p* < 0.001). Although students attending the Control schools also exhibited a long-term gain, it was much smaller (∆*M_BL-FU_* = 1709, *d* = 0.18, *p* < 0.001).

## 4. Discussion

In the present experiment, four elementary schools were randomly assigned to either play the FIT Game or to a no-intervention Control group employing the same data-collection practices. Prior to the intervention, children in the two groups consumed comparable amounts of vegetables at school and had comparable skin-carotenoid concentrations, although children in the Control schools consumed slightly more fruit in the cafeteria. During the intervention phase, children attending the FIT Game schools consumed significantly more vegetables (*d* = 0.41) and fruit (*d* = 0.39) compared to the baseline. This increase in at-school consumption was reflected in their skin carotenoid concentrations (*d* = 0.66), which was also significantly different between the FIT Game and Control schools. Importantly, the significant within-group gain in vegetable consumption was maintained 3 months after the completion of the FIT Game, and the significant increase in skin-carotenoid concentrations provides evidence against good-subjects or reactivity effects. 

These within-group differences replicate prior reports of beneficial effects of the FIT Game [27,29,30]. The uninterrupted implementation of the FIT Game was implemented to encourage the development of habitual patterns of vegetable consumption in the cafeteria. The sustained effect of the intervention at the 3-month follow-up suggests that, indeed, children attending the FIT Game schools got in the habit of eating more vegetables at school. In addition, the sustained increase in skin carotenoid levels suggests that the virtual incentives used in the Game did not negatively impact the intrinsic motivation to eat FV. 

Three limitations of the present study are noteworthy. First, although many students participated, the number of schools assigned to the two groups (FIT Game vs. Control) was limited to two. This provides limited power in a cluster-randomized design and limits replication opportunities. Another related limitation is the complexity of modeling hierarchical data with zero-inflated dependent variables. The current MLM analysis suffers from assumption violations as documented in the residual diagnoses within Data Availability Statement. The potential use of Hurdle and two-stage models could be of use in further experiments despite being applicable for this particular dataset (only four schools).

A second limitation, from a design perspective, is that although the FIT Game only targeted vegetable consumption for change, there were also significant within- and between-group increases in fruit consumption. Anecdotal evidence suggests that some children have difficulty discriminating fruits from vegetables. For example, children occasionally reported doing their best for the FIT heroes by eating all of their oranges or apples, not realizing that the goal was to eat more baby carrots. Although the present findings suggest that the effects of the FIT Game were not confined to the behavior targeted for improvement, the generalization to fruit consumption is a beneficial outcome that likely influenced the within- and between-groups differences in skin carotenoid concentrations. 

A third limitation is that the total increase in FV consumption was modest: An average of +26.45 g in the intervention phase and +5.53 g at the 3-month follow-up. The former of these increases is in the lower range of increases when compared with prior published studies evaluating the FIT Game (range +19.5 to +47.2 g) [27,29,30]. This more modest effect could be due to the different data-collection methods used in the present study—visual estimation of FV consumption from photos of children’s lunch trays. Alternatively, the ~8-week version of the FIT Game may simply have produced more modest increases in FV consumption than the shorter versions used in prior studies. 

To encourage the widespread adoption of the FIT Game in primary and elementary schools, it was designed to be low-cost and low-effort. Virtual incentives earned within the game narrative are cost-free, easier to distribute than tangible incentives, and (in our experience) do not induce cheating or parental concerns about “bribing” children to do things that are in their own best interests. All of these characteristics make the FIT Game ideal for schools that wish to promote healthy eating. 

That said, future research should explore other no-cost, low-effort behavioral technologies that could be combined with the FIT Game to further increase its efficacy. For example, larger effects might be achieved by serving lunch after recess or serving fruit as desert, rather than with lunch [45,46]. Combining the FIT Game with an at-home component may also prove beneficial, as this may further increase the probability that children’s increased willingness to eat FV at school will generalize to the home. 

## Figures and Tables

**Table 1:** Number of participants and demographics of all children attending the four participating schools.

	2016–2017 School Year		2017–2018 School Year	
	**FIT Game**	**Control**	**FIT Game**	**Control**
Participating children (*n*)	326	353	555	625
Female	46%	46%	48%	47%
White	80%	80%	57%	50%
Hispanic	11%	16%	35%	36%
Other	9%	4%	8%	14%
Qualifying for free/reduced lunch *	45%	53%	73%	75%

* US families qualify for free school lunch if their income is ≤130% of the federal poverty line; reduced-price lunch for families ≤ 185% of poverty.

**Table 2:** Parameter estimates for four multilevel models (MLM), one for vegetable and fruit consumption (in grams) each separately, as well as combined (FV), and skin carotenoid counts investigating if the intervention moderated change across each phase of the experiment.

	MLM MODEL 1 Vegetable Consumption (g)		MLM MODEL 2 Fruit Consumption (g)		MLM MODEL 3 Combined FV Consumption (g)		MLM MODEL 4 Skin Carotenoid Concentration	
**Fixed Effects**	**b (SE)**	**Sig.**	**b (SE)**	**Sig.**	**b (SE)**	**Sig.**	**b (SE)**	**Sig.**
Intercept	10.78 (4.07)	0.095	38.26 (5.53)	<0.001 ***	50.90 (11.55)	<0.001 ***	39642 (1792)	<0.001 ***
Grade	1.77 (0.36)	<0.001 ***	2.75 (0.47)	<0.001 ***	−19.79 (2.33)	<0.001 ***	−181 (221)	0.413
Sex: *Male* vs*. Female*							−461 (650)	0.478
Race/Ethnicity (*reference* = *White*)					o			
*Black*							−6861 (1655)	<0.001 ***
*Hispanic*							−982 (931)	0.292
*Asian/Pacific Islander*							559 (2124)	0.792
*American Indian/Alaskan Native*							399 (2123)	0.851
*Prefer not to say*							−1308 (1392)	0.348
Baseline BMI							−520 (101)	<0.001 ***
Exposure to Second-Hand Smoke							−23 (824)	0.978
Intervention								
*FIT Game vs. Control*	0.01 (5.55)	0.999	−3.39 (7.57)	0.675	−3.04 (16.08)	0.867	−141 (797)	0.866
Phase (*reference* = Baseline)								
*Post-Intervention*	1.43 (1.20)	0.234	−4.05 (7.57)	0.035 *	−3.04 (16.08)	0.160	2425 (436)	<0.001 ***
*Follow-up, 3 months*	−2.22 (1.20)	0.064	−15.47 (1.91)	<0.001 ***	−3.30 (2.35)	<0.001 ***	1709 (445)	<0.001 ***
Interaction: Intervention X Phase								
*Post-Intervention*	9.23 (1.77)	<0.001 ***	19.71 (2.82)	<0.001 ***	29.75 (3.47)	<0.001 ***	3681 (659)	<0.001 ***
*Follow-up, 3 months*	7.63 (1.76)	<0.001 ***	18.42 (2.83)	<0.001 ***	25.32 (3.44)	<0.001 ***	3241 (665)	<0.001 ***
**Random Effects**	**Var.**	**Sig.**	**Var.**	**Sig.**			**Var.**	**Sig.**
Schools	28.24	<0.001 ***	52.02	<0.001 ***	249.45	<0.001 ***	78050	0.832
Students within schools	342.31	<0.001 ***	386.56	<0.001 ***	981.03	<0.001 ***	56205291	<0.001 ***
Residual	534.92		1392.04		1931.93		33018162	
**Sample Size**	**n**		**n**		**n**		**n**	
Schools	4		4		4		4	
Students within schools	1640		1640		1575		666	
Residual	4289		4284		4031		1862	
**Model *R*^2^**	**Est.**		**Est.**		**Est.**		**Est.**	
Conditional (Marginal)	0.426 (0.028)		0.273 (0.044)		0.425 (0.060)		0.675 (0.120)	

Note. Each of the four multilevel models (MLM), one for each measure, controlled for grade and the nesting of repeated observation on the child, as well as children nested within the school. The MLM for skin carotenoids additionally controlled for grade, sex, race/ethnic group, baseline body mass index, and child’s exposure to second hand smoke in their home. Estimated marginal means with standard errors for the means (SEM) are presented in Table 3. Significance (Sig.) of fixed model parameter estimates via the Wald t-test utilizing Satterthwaite’s method for degrees of freedom and Likelihood Ratio Test for single term deletion of random effects for significance of estimated variance components (Var.). The marginal *R*^2^ considers only the variance of the fixed effects, while the conditional *R*^2^ takes both the fixed and random effects into account. * *p* < 0.05. *** *p* < 0.001.

**Table 3:** Estimated group marginal means (±SEM) vegetable and fruit consumption (in grams) and skin carotenoid counts in each phase of the experiment with between-group comparisons across the phase of the experiment.

Phase	Measure	FIT Game	Control	Sig.
Baseline	Vegetable	15.96 (3.94)	15.95 (3.91)	0.999
	Fruit	42.90 (5.37)	46.29 (5.33)	0.654
	FV	60.22 (11.38)	63.53 (11.35)	0.850
	Carotenoids	29,062 (588)	29,203 (534)	0.904
Intervention	Vegetable	26.61 (3.94)	17.38 (3.92)	0.096
	Fruit	58.57 (5.37)	42.24 (5.34)	0.031 *
	FV	86.93 (11.39)	60.22 (11.36)	0.097
	Carotenoids	35,168 (596)	31,628 (543)	0.007 *
Follow-up	Vegetable	21.36 (3.94)	13.73 (3.92)	0.169
	Fruit	45.85 (5.37)	30.82 (5.34)	0.047 *
	FV	66.01 (11.38)	43.74 (11.35)	0.166
	Carotenoid	34,012 (600)	30,912 (550)	0.015 *

Note. Each of the four multilevel models (MLM), one for each measure, controlled for grade and the nesting of repeated observation on the child, as well as the children nested within the school. The MLM for skin carotenoids additionally controlled for grade, sex, race/ethnic group, baseline body mass index, and child’s exposure to second hand smoke in their home. Full model specifications and parameter estimates are presented in Table 2. Graphical representation of the estimated marginal means is presented in Figure 3. Significance (Sig.) is given for post hoc pairwise t-test comparisons (unadjusted) between the FIT Game and the Control condition means. FV: Combined fruit and vegetables. * *p* < 0.05.

## Data Availability

Full R code, data, and output are available on Open Science Framework at doi: 10.17605/OSF.IO/GVPQS.
